# Medication background of Japanese men undergoing prostate needle biopsy: A medication profile based on the number of medications and drug classes

**DOI:** 10.1371/journal.pone.0354283

**Published:** 2026-10-29

**Authors:** Kazuki Sato, Takashi Kawahara, Akihito Hashizume, Masanobu Yamazaki, Hiroki Ito, Teppei Takeshima, Shinnosuke Kuroda, Hiroji Uemura, Kazuhide Makiyama, Jun-ichi Teranishi

**Affiliations:** 1 Departments of Urology and Renal Transplantation, Yokohama City University Medical Center, Yokohama, Japan; 2 Department of Urology, Yokohama City University, Graduate School of Medicine, Yokohama, Japan; Morehouse School of Medicine, UNITED STATES OF AMERICA

## Abstract

Prostate cancer is common in older men, and the treatment decision depends strongly on the patient’s general condition and comorbidities. However, the medication background of men who reach prostate biopsy has rarely been described. We used a large Japanese administrative claims database (Medical Data Vision) to profile the medications of men who underwent prostate needle biopsy between April 2008 and June 2019. We examined the number of medications and the drug classes in relation to body mass index (BMI) and age. We identified 67,812 men, all aged 60 years or older. The mean number of medications was lowest at BMI 20–25 and increased toward both low and high BMI, showing a U-shaped pattern (minimum 1.44 drugs at BMI 22.0–22.5); the increase was driven mainly by high BMI. This pattern was consistent across age strata, and in a negative binomial regression adjusted for age the quadratic term of BMI remained significant (p < 0.001), with an adjusted minimum at BMI 20.5. The composition of drug classes differed by body size: metabolic drugs (antihypertensive/cardiovascular, dyslipidemia, diabetes, and hyperuricemia drugs) were more common at high BMI, whereas gastrointestinal and analgesic drugs were relatively more common at low BMI, and all classes increased with age. In conclusion, among men undergoing prostate biopsy, the number of medications was lowest at BMI 20–25 and the qualitative medication profile differed by BMI. These findings describe the medication background of this population and may inform the design of future risk-stratified or prospective studies.

## Introduction

Prostate cancer is one of the most common cancers in older men, and its incidence is increasing as the population age [1]. Many prostate cancers grow slowly. They do not always need immediate radical treatment, and active surveillance is an accepted option for low-risk disease [2]. For each patient, we must choose between radical treatment and active surveillance or coexistence. This decision does not depend only on the cancer itself. It also depends strongly on the patient’s general condition and comorbidities, especially in older men [3]. Therefore, it is clinically important to understand the general background of patients who are suspected of prostate cancer and undergo further examination.

One simple way to capture this background is the patient’s medications. Polypharmacy is common in older people, and the number of medications reflects the burden of comorbidities [4]. Polypharmacy is also related to adverse outcomes and prognosis [5]. In addition, the drug classes that a patient takes may reflect the types of underlying diseases more specifically. This medication information is almost always recorded in daily practice, and it can be assessed at no extra cost. Thus, the medication background, which combines the number of medications (quantity) and the drug classes (quality), can be a practical framework to describe the patient in the setting of prostate cancer care.

On the other hand, the diseases behind polypharmacy are diverse. They include autoimmune diseases, lifestyle-related diseases, and malignancies, and their composition and clinical meaning differ by population. Therefore, findings from the general population cannot be applied directly to each clinical setting, and polypharmacy should be evaluated within the population of interest. Urologists must decide whether prostate cancer is present in older men who undergo prostate biopsy, and it is meaningful to understand the medication background of this specific population. However, few studies have described the number of medications and drug classes in relation to body mass index (BMI) and age in this population in Japanese real-world practice. In this study, we described the medication background of men undergoing prostate needle biopsy, in terms of the number of medications and drug classes, using a large Japanese database.

## Materials and methods

### Study design and data source

This was a retrospective study using the Medical Data Vision (MDV) database, a large Japanese administrative claims database. We extracted men who underwent prostate needle biopsy between April 2008 and June 2019. For each patient, we used the record at the time of biopsy. The analytic dataset contained one record per patient (each patient identifier appeared once), so repeat biopsies in the same man did not contribute more than one observation. All men were aged 60 years or older (range 60–99). The study was approved by the Institutional Review Board of Yokohama City University Medical Center, Yokohama, Japan (approval number F240500030). Because this was a retrospective analysis of anonymized claims data, the requirement for informed consent was waived by the review board. The dataset was originally extracted from the MDV database on August 17, 2020, for a broader research program. For the present study, the data were accessed for research purposes on May 30, 2024, after ethics approval. The authors did not have access to any information that could identify individual participants during or after data collection, because the database was fully anonymized.

### Variables

We collected age, body mass index (BMI), and the availability and value of prostate-specific antigen (PSA). BMI values below 10 or above 60 kg/m^2^ were regarded as implausible and were excluded from BMI-related analyses. For each patient, we counted the number of prescribed drugs (the number of medications). Patients with no recorded medication were treated as taking zero drugs. The extract contained the set of prescribed drugs recorded around the time of biopsy but did not include dispensing dates, so we could not define a fixed look-back or look-forward window, distinguish active from past prescriptions, or separate short-course from chronic therapy; each distinct drug was counted once per patient, and fixed-dose combination products were counted as their individual components.

### Drug classification

We classified each prescribed drug into a therapeutic class based on its generic name (for example, antihypertensive/cardiovascular, dyslipidemia, diabetes, hyperuricemia/gout, gastrointestinal, antithrombotic, psychotropic/sleep, analgesic/anti-inflammatory, urological, and antibiotic); the classes and their representative drugs are shown in S1 Table. Each drug was assigned to a single class. Some drugs have more than one common indication. For such drugs, we assigned the class that matched the predominant use in this population. For example, uroselective α1-blockers (silodosin, tamsulosin, and naftopidil) were classified as urological, whereas doxazosin was classified as antihypertensive/cardiovascular, and low-dose aspirin was classified as antithrombotic. A patient was regarded as using a drug class if he took at least one drug in that class. Because the classification was based on drug names, the prevalence of a drug class approximates, but does not equal, the prevalence of the corresponding disease. Diagnosis (disease) codes were not included in our data extract; therefore, we could not identify comorbidities directly or construct a validated claims-based comorbidity index, and we used drug classes only as an approximate marker of the underlying conditions. Each therapeutic class was mapped to the corresponding Anatomical Therapeutic Chemical (ATC) groups so that the assignments could be audited against a published standard (S5 Table). Drugs with more than one plausible indication accounted for 4.2% of all prescriptions; in a sensitivity analysis we reassigned the two most ambiguous of these (low-dose aspirin from antithrombotic to analgesic, and doxazosin from antihypertensive/cardiovascular to urological) and recomputed the class composition.

### Statistical analysis

We described patient characteristics for the whole cohort and by biopsy result. Continuous variables were compared with the Mann–Whitney U test, and categorical variables with the chi-square test. We examined the number of medications by BMI, using both five BMI categories and fine 0.5-unit bins. To test whether this relationship was explained by age, we fitted a negative binomial regression for the number of medications with age, BMI, and BMI^2^ as covariates; the negative binomial model was chosen because the medication count was overdispersed. We estimated the BMI at which the adjusted count was lowest and obtained a bootstrap 95% confidence interval (1,000 resamples) for it. We also modeled BMI with a restricted cubic spline, reported incidence rate ratios with 95% confidence intervals, quantified overdispersion with the Poisson dispersion statistic, and analyzed the number of medications among users (patients taking at least one drug) separately. For each drug class, we compared its prevalence across BMI groups (<20, 20–25 [reference], and ≥25 kg/m^2^) using logistic regression adjusted for age, reported odds ratios with 95% confidence intervals, and applied a Bonferroni correction for the 20 comparisons (ten classes × two BMI groups). In a sensitivity analysis, we recomputed the medication-count analysis after excluding urological drugs, which may be related to biopsy referral. We also described the prevalence of each drug class across a 3 × 3 grid of age (<70, 70–79, and ≥80 years) and BMI (<20, 20–25, and ≥25 kg/m^2^). All analyses were performed with Python (pandas and statsmodels). A two-sided p value below 0.05 was considered significant.

## Results

In this study, we described the medication background of Japanese men who underwent prostate needle biopsy for suspected prostate cancer, using the MDV database. We identified 67,812 men, all aged 60 years or older. PSA values were available in only 5,044 men (7.4%). The baseline characteristics are shown in Table 1. After excluding 5,378 men with missing and 129 with implausible BMI, 62,305 men remained for the BMI-related analyses; the cohort assembly, with the total number of biopsies before exclusions and a comparison of included and excluded men, is shown in S1 Fig. BMI was missing more often among men with a measured PSA than among those without (31.8% versus 6.2%), probably reflecting different recording of height and weight at the encounters where PSA was measured. Overall, 31,876 men (51.2%) had no recorded medication, and about 16% of prescriptions were not assigned to any of the ten drug classes.

**Table 1:** Baseline characteristics of the biopsy cohort.

Characteristic	Overall (n = 67,812)
Age, years, mean ± SD	73.0 ± 6.5
BMI, kg/m², mean ± SD	23.5 ± 3.0
BMI category, n (%)	
<18.5	4.3
18.5–22	26.1
22–25	40.9
25–30	26.3
≥30	2.4
Medication use, n (%)	47.5
Medications (all), median (IQR)	0 (0–2)
Medications (users), mean ± SD	3.3 ± 2.9
PSA measured, n (%)	7.4

*Values are for the whole cohort. BMI was analyzed after excluding implausible values (valid n = 62,305); BMI category percentages are among men with valid BMI. IQR, interquartile range; PSA, prostate-specific antigen; SD, standard deviation.*

The number of biopsies peaked at 70–74 years and declined at the oldest ages (Fig 1).

**Fig 1:**
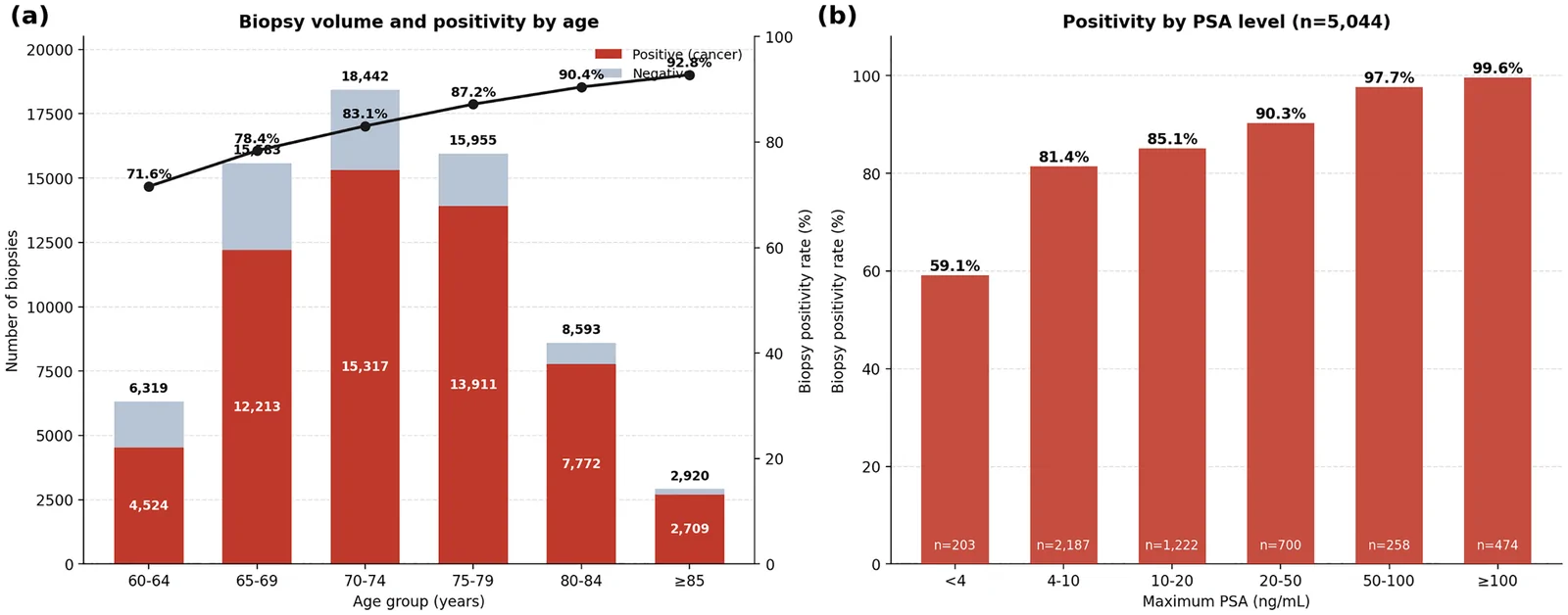
Prostate biopsies by age group. Number of biopsies in each 5-year age group (N = 67,812).

In the whole cohort, the mean number of medications showed an apparent U-shape across BMI, lowest in the middle and higher at both ends (Fig 2A, S2 Table). The mean number was lowest in the BMI 22–25 category (1.52 drugs) and higher in both the low-BMI group (<18.5: 1.73 drugs) and the obese group (≥30: 2.23 drugs), and the descriptive minimum in the finer 0.5-unit analysis was again at BMI 22.0–22.5 (1.44 drugs). This apparent U-shape was asymmetric in origin. Among men taking at least one drug, the mean number of medications was almost flat across the lower BMI range and rose only at high BMI (Fig 2B: < 18.5, 3.29; 22–25, 3.22; ≥ 30, 4.21), whereas the proportion of men taking any drug was higher at both BMI extremes (52.6% at <18.5 and 52.9% at ≥30 versus 47.3% at 22–25; red line in Fig 2A). The higher medication burden at low BMI therefore reflected mainly a larger proportion of medicated men rather than more drugs per user, whereas at high BMI both the proportion and the number of drugs per user increased. This pattern was not explained by age; although the number of medications increased with age, the U-shaped minimum near BMI 20–25 was preserved in every age stratum (Fig 3A). After adjustment for age in a negative binomial regression, the quadratic term of BMI remained significant (p < 0.001) and the adjusted minimum was at BMI 20.5 (bootstrap 95% CI 19.4–21.3), and a restricted cubic spline gave a similar shape (Fig 3B, S3 Table). Because the medication count was overdispersed (Poisson dispersion statistic, 4.01), a negative binomial model was used; the incidence rate ratio was 0.86 (95% CI 0.84–0.89) for BMI and 1.004 (1.003–1.004) for BMI^2^ (both p < 0.001). The adjusted rate was 1.40 times higher at BMI 30 than at the minimum but only 1.02 times higher at BMI 18, confirming that the increase in the number of medications was driven mainly by high BMI.

**Fig 2:**
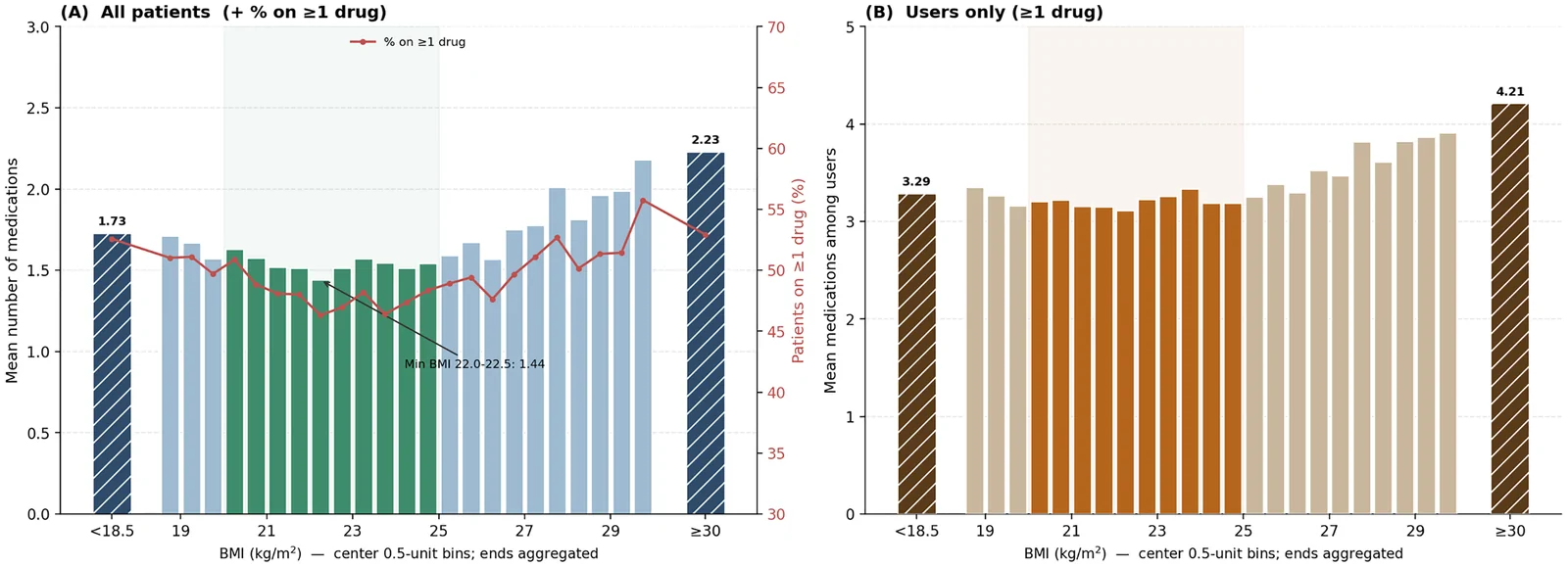
Number of medications by BMI. (A) Mean number of medications in all patients by BMI in the biopsy cohort (center bars, 0.5-unit bins; end bars, aggregated <18.5 and ≥30). The number of medications was lowest at BMI 20–25 (1.44 at BMI 22.0–22.5) and higher at both extremes, showing an apparent U-shape. The red line shows the percentage of patients taking at least one drug (right axis). (B) Mean number of medications among users (patients taking at least one drug), which is flat at low BMI and rises only at high BMI, indicating that the low-BMI increase reflects a higher proportion of medicated men rather than more drugs per user. Error bars, 95% confidence interval.

**Fig 3:**
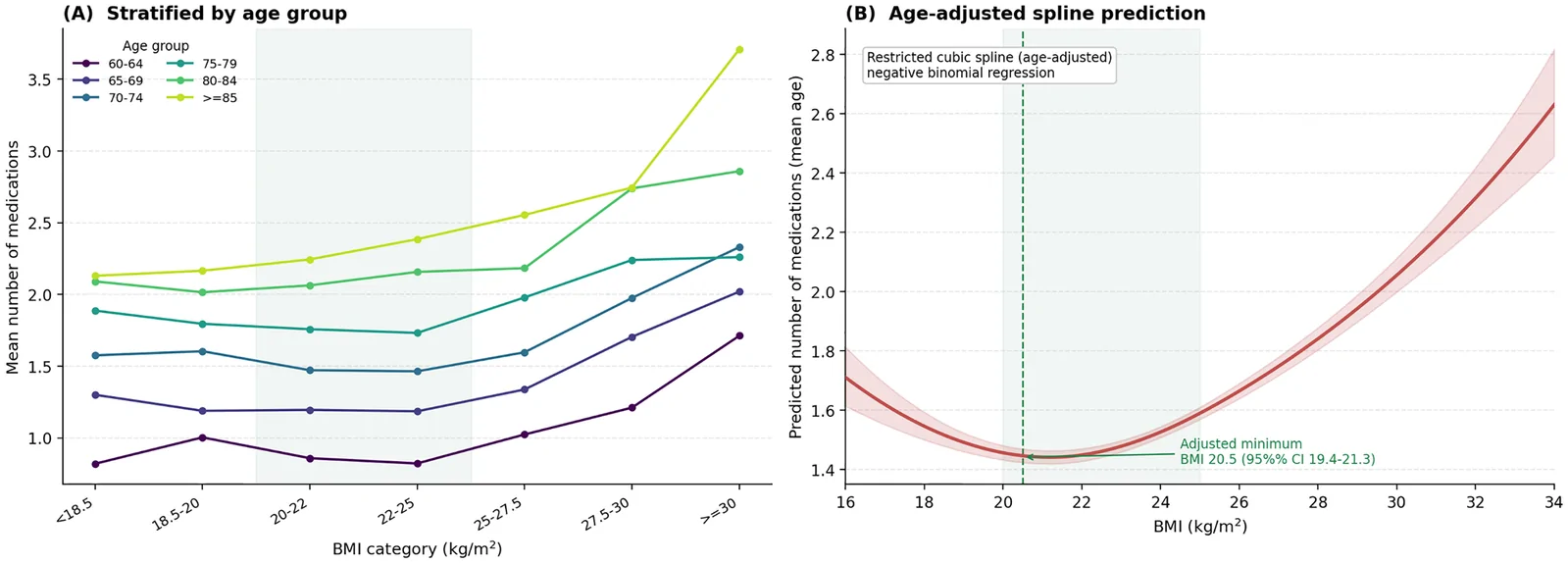
The U-shaped association persists after age adjustment. (A) BMI and the mean number of medications by age group; the minimum near BMI 20–25 was seen in every age group. (B) Age-adjusted predicted number of medications (negative binomial regression); the quadratic term of BMI was significant (p < 0.001) and the adjusted minimum was at BMI 20.5.

The composition of drug classes differed by body size (Fig 4, S1 Table). Gastrointestinal (23.6%), urological (20.8%), and antihypertensive/cardiovascular (16.1%) drugs were the most common overall. Across the age × BMI grid, the metabolic classes—antihypertensive/cardiovascular, dyslipidemia, diabetes, and hyperuricemia drugs—became more common as BMI increased, whereas gastrointestinal and analgesic drugs were relatively more common at low BMI; gastrointestinal drugs and antibiotics were elevated at both BMI extremes, and all classes became more common with age. For example, the prevalence of antihypertensive/cardiovascular drugs rose from 13% at BMI < 20–21% at BMI ≥ 25. The U-shaped pattern of medication count therefore reflected different drug compositions at the two extremes, with metabolic drugs predominating at high BMI and gastrointestinal and analgesic drugs at low BMI. These differences were confirmed in age-adjusted logistic regression with BMI 20–25 as the reference (S4 Table): for the metabolic classes the odds of drug use fell at low BMI and rose at high BMI (antihypertensive/cardiovascular, odds ratio 0.79 [95% CI 0.73–0.85] at BMI < 20 and 1.46 [1.39–1.53] at BMI ≥ 25; dyslipidemia, 0.57 [0.51–0.64] and 1.43 [1.35–1.52]; hyperuricemia, 0.64 [0.56–0.75] and 1.65 [1.53–1.79]), whereas gastrointestinal and analgesic drugs were more common at low BMI (odds ratios at BMI < 20, 1.13 [1.06–1.19] and 1.33 [1.18–1.49]). Urological drugs changed little across BMI groups (odds ratios 1.05 and 1.06; not significant after Bonferroni correction). The other associations reported here remained significant after Bonferroni correction for 20 comparisons. Drugs with more than one plausible indication accounted for only 4.2% of all prescriptions, and the class composition was robust to the assignment rule: under the alternative rule (S5 Table) the prevalence of every class was essentially unchanged except for the two deliberately reassigned classes (antithrombotic, 6.0% to 3.9%, and analgesic, 3.9% to 6.7%, when aspirin was reclassified), while the metabolic classes and the BMI gradient were unaffected.

**Fig 4:**
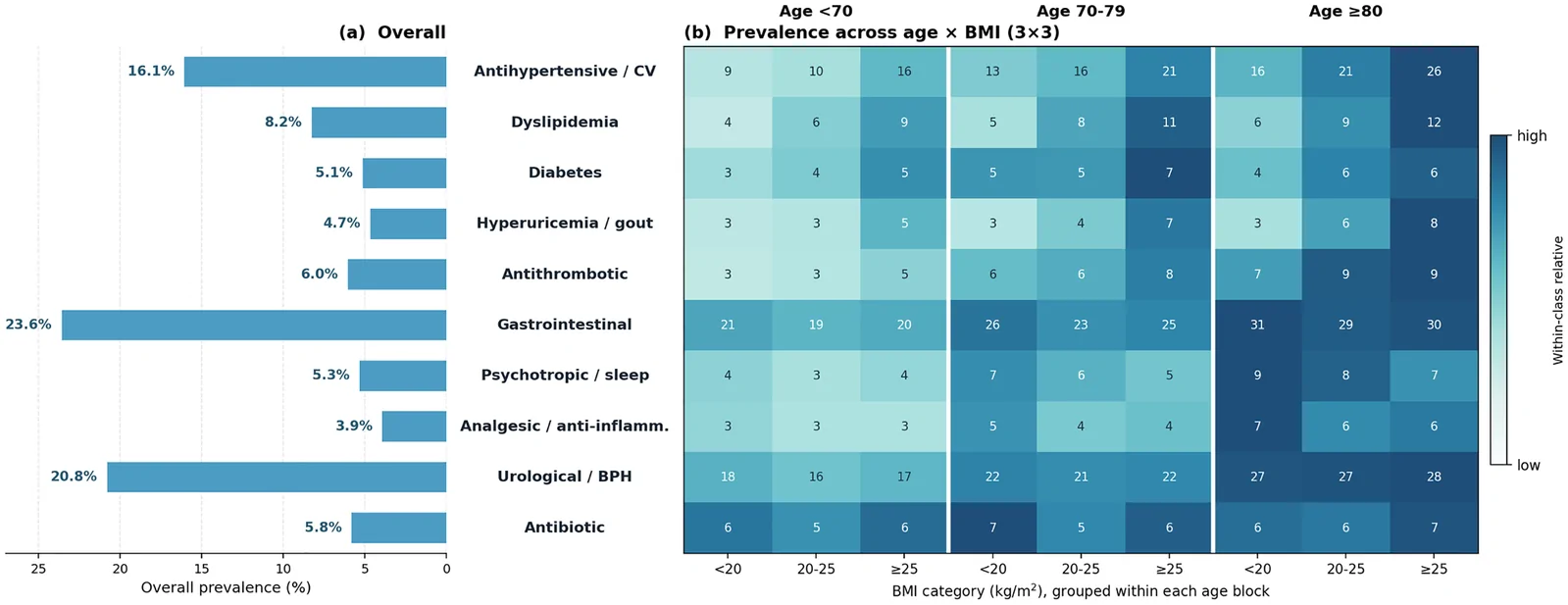
Drug classes in the biopsy cohort. (A) Overall prevalence of each drug class. (B) Prevalence of each drug class across the nine age × BMI cells; color is scaled within each class and the numbers are the absolute prevalence (%). Metabolic classes increased with BMI, whereas gastrointestinal and analgesic drugs were relatively more common at low BMI, and all classes increased with age.

only age (odds ratio 2.00 [95% CI 1.70–2.35]) and PSA (2.12 [1.88–2.39] per log unit) Excluding urological drugs, which may relate to biopsy referral, did not change the medication findings: the number of medications remained lowest at BMI 22–25 and higher at both extremes.

## Discussion

In this study, we profiled the medications of a cohort of patients undergoing prostate needle biopsy. The number of medications was lowest at BMI 20–25 and increased at both the low-BMI and high-BMI ends, showing a U-shaped pattern driven mainly by the increase at high BMI. This minimum was consistent across age strata; in every age group, the number of medications was lowest around BMI 20–25. When we looked at drug classes, the overall drug burden increased with age. Within this, obese patients had a higher proportion of metabolic drugs (antihypertensive/cardiovascular, dyslipidemia, and hyperuricemia drugs), whereas lean patients had a higher proportion of gastrointestinal and analgesic drugs; gastrointestinal drugs and antibiotics were elevated at both BMI extremes. Urological drugs changed little with BMI, and gastrointestinal drugs were slightly more common at low BMI. Thus, even within the same age group, the qualitative medication profile differed by BMI.

Polypharmacy is common in older people [4], and it is associated with many adverse outcomes, such as falls, adverse drug events, and hospitalization [5]. The incidence of prostate cancer increases with age [1], and most men who undergo biopsy are elderly. As the number of medications increases with age, this problem becomes more important. However, few studies have described the medication profile at the time of prostate biopsy. In our cohort, 47.5% of patients were taking at least one drug, users took a mean of 3.3 drugs, and 12.2% were taking five or more drugs and 2.1% ten or more drugs, the conventional thresholds for polypharmacy and hyperpolypharmacy.

Prostate cancer often allows long survival even in the metastatic setting [6], so men who reach biopsy may live for years and undergo prolonged treatment. During such treatment, androgen-deprivation and related therapies carry systemic risks—for example, gonadotropin-releasing hormone agonists have been associated with more cardiovascular events than the antagonist degarelix [7,8], and long-term androgen deprivation increases fracture risk [9]. Because the tolerability of these treatments depends on the patient’s general background, describing the medication background at the time of biopsy provides a rationale for future studies linking baseline comorbidity burden to treatment tolerability and outcomes; our cross-sectional data cannot themselves establish such links.

This study has several limitations. First, it did not include information on prostate cancer stage or grade, so we could not evaluate the relationship with disease extent. Second, our analysis was based only on the medication profile and does not directly show the actual comorbidities. Because diagnosis codes were not included in our extract, we could not construct a validated comorbidity index, and the medication-based classification is only an approximation. We did not measure frailty, nutritional, or functional status; occult illness or advanced disease could equally explain both a lower BMI and a higher medication count, and our cross-sectional design cannot distinguish these possibilities. Third, this was a retrospective analysis of a single administrative database, and we cannot infer causal relationships. Fourth, PSA was available in only 7.4% of the cohort, so it could not be used in the main analyses; the biopsy-only design also means the cohort is itself selected. Fifth, the medication exposure window could not be defined precisely: the extract did not contain dispensing dates, so we could not specify a look-back interval, separate active from past or short-course from chronic prescriptions, or exclude drugs that might have been started in response to the biopsy result. Medication counts should therefore be read as the drugs recorded around the time of biopsy rather than as a validated point-prevalence measure. In addition, the database captures only prescribing within participating facilities, so drugs obtained elsewhere and over-the-counter products were not observed, which may contribute to the low mean number of medications.

## Conclusion

In conclusion, we described the medication profile of Japanese men with suspected prostate cancer. The number of medications was lowest at BMI 20–25. Even within the same age stratum, the medication profile differed by BMI: metabolic drugs were more common at high BMI and gastrointestinal and analgesic drugs at low BMI, whereas some classes such as urological drugs changed little. These findings describe the medication background of men who undergo prostate biopsy and provide a descriptive profile of this biopsy-only cohort, which is subject to selection bias; they may inform the design of future risk-stratified or prospective studies rather than current clinical care.

## Supporting information

S1 Fig Participant flow diagram. (TIF)

S1 Table Drug classes and representative drugs. For each therapeutic class, the overall prevalence and the most frequent generic drugs. Each drug was assigned to a single class by its predominant use, and a patient was regarded as using a drug class if he took at least one drug in that class.
(DOCX)

S2 Table Number of medications by BMI category. n, mean number of medications (±SD), and median for each of the five BMI categories. (DOCX)

S3 Table Negative binomial regression for the number of medications, adjusted for age. (DOCX)

S4 Table Age-adjusted odds ratios for each drug class by BMI group. Odds ratios (95% CI) for the use of each drug class at BMI < 20 and ≥25 kg/m^2^, with BMI 20–25 as the reference, adjusted for age; p-values are Bonferroni-corrected for 20 comparisons.
(DOCX)

S5 Table Mapping of drug classes to ATC groups and sensitivity to the assignment rule. Each therapeutic class with its corresponding Anatomical Therapeutic Chemical (ATC) groups, the overall prevalence under the primary assignment, and the prevalence under an alternative rule (low-dose aspirin reclassified as analgesic and doxazosin as urological).
(DOCX)

S1 File Raw data for the tables and figures. De-identified analytic dataset (Sheet 1) and the full drug-to-class mapping (Sheet 2).
(XLSX)

S2 File Analysis code. Python code (with package versions) used to classify drugs and to perform all analyses.
(ZIP)
