# Supplementary material for: Medication background of Japanese men undergoing prostate needle biopsy: A medication profile based on the number of medications and drug classes
Source: PLoS One. 2026 Oct 29;21(10):e0354283. doi: 10.1371/journal.pone.0354283 (PMC13718932; doi:10.1371/journal.pone.0354283)
Supplement: Supplementary file 1 [file pone.0354283.s002.docx]

**S1 Table. Drug classes and representative drugs.**

| **Drug class** | **Prevalence** | **Representative drugs (most frequent)** |
| --- | --- | --- |
| Antihypertensive / cardiovascular | 16.1% | amlodipine, nifedipine, olmesartan, carvedilol, candesartan |
| Dyslipidemia | 8.2% | rosuvastatin, atorvastatin, pitavastatin, pravastatin, ezetimibe |
| Diabetes | 5.1% | sitagliptin, metformin, glimepiride, voglibose, linagliptin |
| Hyperuricemia / gout | 4.7% | febuxostat, allopurinol, benzbromarone, topiroxostat, colchicine |
| Antithrombotic | 6.0% | aspirin, clopidogrel, warfarin, cilostazol, rivaroxaban |
| Gastrointestinal | 23.6% | sennoside, magnesium oxide, lansoprazole, esomeprazole, rebamipide |
| Psychotropic / sleep | 5.3% | brotizolam, zolpidem, etizolam, zopiclone, donepezil |
| Analgesic / anti-inflammatory | 3.9% | loxoprofen, pregabalin, prednisolone, celecoxib, acetaminophen |
| Urological / BPH | 20.8% | silodosin, tamsulosin, naftopidil, dutasteride, bicalutamide |
| Antibiotic | 5.8% | levofloxacin, sitafloxacin, clarithromycin, cefcapene pivoxil |

*Each drug was assigned to a single class by its predominant use in this population, and a patient was regarded as using a drug class if he took at least one drug in that class; only the most frequent drugs are shown. Class prevalence approximates, but does not equal, disease prevalence.*
