# Supplementary material for: Medication background of Japanese men undergoing prostate needle biopsy: A medication profile based on the number of medications and drug classes
Source: PLoS One. 2026 Oct 29;21(10):e0354283. doi: 10.1371/journal.pone.0354283 (PMC13718932; doi:10.1371/journal.pone.0354283)
Supplement: Supplementary file 2 [file pone.0354283.s003.docx]

**S2 Table. Number of medications and biopsy positivity by BMI category.**

| **BMI category (kg/m²)** | **n** | **Mean medications (±SD)** | **Median** | **Positivity rate** |
| --- | --- | --- | --- | --- |
| <18.5 | 2,703 | 1.73 ± 2.61 | 1 | 84.8% |
| 18.5–22 | 16,270 | 1.58 ± 2.53 | 0 | 82.9% |
| **22–25** | **25,505** | **1.52 ± 2.50** | **0** | **83.0%** |
| 25–30 | 16,359 | 1.74 ± 2.76 | 0 | 83.2% |
| ≥30 | 1,468 | 2.23 ± 3.31 | 1 | 81.9% |

*The number of medications was lowest at BMI 22–25 (U-shaped), whereas the biopsy positivity rate was essentially constant across BMI categories (81.9–84.8%, p=0.11). SD, standard deviation.*
