# Supplementary material for: Medication background of Japanese men undergoing prostate needle biopsy: A medication profile based on the number of medications and drug classes
Source: PLoS One. 2026 Oct 29;21(10):e0354283. doi: 10.1371/journal.pone.0354283 (PMC13718932; doi:10.1371/journal.pone.0354283)
Supplement: Supplementary file 3 [file pone.0354283.s004.docx]

**S3 Table. Multivariable regression analyses.**

**A. Negative binomial regression — outcome: number of medications (n = 62,305)**

| **Predictor** | **IRR / Coefficient** | **95% CI** | **p** |
| --- | --- | --- | --- |
| Age (per 1 year) | IRR 1.039 | 1.038 to 1.041 | <0.001 |
| BMI (per 1 unit) | IRR 0.861 | 0.838 to 0.886 | <0.001 |
| **BMI² (quadratic term)** | **IRR 1.0037** | **1.0031 to 1.0042** | **<0.001** |

*Age-adjusted minimum number of medications at BMI 20.5 kg/m² (bootstrap 95% CI 19.4–21.3). Poisson dispersion statistic 4.01 confirmed overdispersion. A restricted cubic spline gave a similar shape. IRR, incidence rate ratio; CI, confidence interval.*

**B. Multivariable logistic regression — outcome: biopsy positivity (n = 62,305)**

| **Predictor** | **Odds ratio** | **95% CI** | **p** |
| --- | --- | --- | --- |
| **Age (per 10 years)** | **2.04** | **1.97 to 2.12** | **<0.001** |
| BMI (per 5 units) | 1.07 | 1.03 to 1.11 | <0.001 |
| Medications (per 5 drugs) | 0.87 | 0.84 to 0.91 | <0.001 |

*Only age was independently associated with biopsy positivity; odds ratios for BMI and medication count were close to 1. In the 65–75-year subgroup: age 1.93 (1.76–2.10), BMI 1.08 (1.03–1.13), medications 0.83 (0.79–0.88). CI, confidence interval.*
