# Supplementary material for: Medication background of Japanese men undergoing prostate needle biopsy: A medication profile based on the number of medications and drug classes
Source: PLoS One. 2026 Oct 29;21(10):e0354283. doi: 10.1371/journal.pone.0354283 (PMC13718932; doi:10.1371/journal.pone.0354283)
Supplement: Supplementary file 4 [file pone.0354283.s005.docx]

**S4 Table. Age-adjusted odds ratios for each drug class by BMI group.**

| **Drug class** | **BMI <20 vs 20–25 OR (95% CI)** | **BMI ≥25 vs 20–25 OR (95% CI)** |
| --- | --- | --- |
| Antihypertensive / cardiovascular | 0.79 (0.73–0.85)* | 1.46 (1.39–1.53)* |
| Dyslipidemia | 0.57 (0.51–0.64)* | 1.43 (1.35–1.52)* |
| Diabetes | 0.88 (0.78–0.99) | 1.39 (1.28–1.50)* |
| Hyperuricemia / gout | 0.64 (0.56–0.75)* | 1.65 (1.53–1.79)* |
| Antithrombotic | 0.82 (0.73–0.91)* | 1.26 (1.17–1.35)* |
| Gastrointestinal | 1.13 (1.06–1.19)* | 1.07 (1.03–1.12)* |
| Psychotropic / sleep | 1.16 (1.04–1.28) | 0.93 (0.85–1.00) |
| Analgesic / anti-inflammatory | 1.33 (1.18–1.49)* | 1.07 (0.98–1.18) |
| Urological / BPH | 1.05 (0.99–1.11) | 1.06 (1.02–1.11) |
| Antibiotic | 1.18 (1.07–1.31)* | 1.19 (1.10–1.28)* |

*Odds ratios (95% CI) from logistic regression of each drug class on BMI group, adjusted for age; BMI 20–25 kg/m² is the reference. * significant after Bonferroni correction for 20 comparisons (ten classes × two BMI groups; corrected α = 0.0025). OR, odds ratio; CI, confidence interval.*
