# Supplementary material for: Medication background of Japanese men undergoing prostate needle biopsy: A medication profile based on the number of medications and drug classes
Source: PLoS One. 2026 Oct 29;21(10):e0354283. doi: 10.1371/journal.pone.0354283 (PMC13718932; doi:10.1371/journal.pone.0354283)
Supplement: Supplementary file 5 [file pone.0354283.s006.docx]

**S5 Table. Mapping of drug classes to ATC groups and sensitivity to the assignment rule.**

| **Drug class** | **ATC group(s)** | **Primary %** | **Alt-rule %** |
| --- | --- | --- | --- |
| Antihypertensive / cardiovascular | C01, C02, C03, C07, C08, C09 | 16.1 | 16.0 |
| Dyslipidemia | C10 | 8.2 | 8.2 |
| Diabetes | A10 | 5.1 | 5.1 |
| Hyperuricemia / gout | M04 | 4.7 | 4.7 |
| Antithrombotic | B01 | 6.0 | 3.9 |
| Gastrointestinal | A02, A03, A06, A07, A09 | 23.6 | 23.6 |
| Psychotropic / sleep | N05, N06 | 5.3 | 5.3 |
| Analgesic / anti-inflammatory | N02, M01, H02 | 3.9 | 6.7 |
| Urological / BPH | G04, L02 | 20.8 | 21.3 |
| Antibiotic | J01 | 5.8 | 5.8 |

*ATC, Anatomical Therapeutic Chemical classification. Primary %, prevalence under the primary assignment rule; Alt-rule %, prevalence when low-dose aspirin was reclassified from antithrombotic to analgesic and doxazosin from antihypertensive/cardiovascular to urological. Drugs with more than one plausible indication accounted for 4.2% of all prescriptions.*
