# Supplementary material for: Medication background of Japanese men undergoing prostate needle biopsy: A medication profile based on the number of medications and drug classes
Source: PLoS One. 2026 Oct 29;21(10):e0354283. doi: 10.1371/journal.pone.0354283 (PMC13718932; doi:10.1371/journal.pone.0354283)
Supplement: Supplementary file 6 [file pone.0354283.s010.docx]

**Response to Reviewers**

Manuscript PONE-D-26-33559

Medication background of Japanese men undergoing prostate needle biopsy: a comorbidity profile based on the number of medications and drug classes

We thank the editor and both reviewers for their careful and constructive assessment. We have revised the manuscript substantially, including several new analyses. Below we respond to each point in turn and indicate where the corresponding change appears in the revised manuscript. Revisions are shown in the marked-up manuscript; line numbers refer to the clean revised version. New Supporting Information items (S1 Fig; S4, S5, and S6 Tables) have been added.

**Editor — general guidance**

**Novelty/impact (raised by Reviewer #2).** *Whether the findings advance understanding of prostate cancer development.*

**Response:** We note the editor's guidance that PLOS ONE does not assess novelty or impact, and we have not framed the paper around advancing the understanding of prostate cancer development. The study is presented as a descriptive profile of the medication background of men undergoing prostate biopsy.

**Diagnosis data — settle directly.** *Reviewer #2 states diagnosis data are unavailable; Reviewer #1 assumes a claims-based comorbidity index could be built.*

**Response:** We have settled this directly. Our extract from the Medical Data Vision (MDV) database did not include diagnosis (disease) codes; it contained the prescribed drug names, demographics, BMI, and PSA availability/value only. We therefore could not identify comorbidities directly or construct a validated claims-based comorbidity index (e.g., a Charlson or Elixhauser index), and we used drug classes as an approximate marker of the underlying conditions. This is now stated explicitly in the Methods (Drug classification) and in the Limitations.

**Editor — specific points**

**1. Biopsy positivity definition.** *State whether men with a cancer code before/at biopsy were retained, how repeat biopsies were handled, and whether the definition was validated against pathology.*

**Response:** Positivity was defined from the claims-based prostate cancer classification recorded in the database and was not validated against pathology reports, which were not available. Men with a prostate cancer code recorded before biopsy were not separately excluded. The analytic dataset contains one record per patient (each identifier appears once; no patient appears in both groups), so repeat biopsies do not contribute more than one observation. We now state this in the Methods (Study design and data source) and add, in the Limitations, that because the case definition is unvalidated, the finding that positivity is unrelated to BMI and to the number of medications cannot be fully separated from that definition.

**2. What diagnosis data MDV contains.** *State whether diagnosis information was available and unused, or genuinely absent.*

**Response:** As above, diagnosis codes were genuinely absent from our extract. We state that they were not included, that a validated comorbidity index therefore cannot be constructed, and that drug class was used as an approximation (Methods, Drug classification; Limitations).

**3. Justify and test the drug classification (ATC; multi-indication drugs; alternative rule).** *Map classes to ATC; report how many drugs had more than one plausible indication; rerun under an alternative assignment rule.*

**Response:** We mapped each therapeutic class to the corresponding ATC groups (new S6 Table) so the assignments can be audited against a published standard. Drugs with more than one plausible indication accounted for only 4.2% of all prescriptions. In a sensitivity analysis using an alternative rule (low-dose aspirin reassigned to analgesic, doxazosin to urological), the prevalence of every class was essentially unchanged except for the two deliberately reassigned classes (antithrombotic 6.0%→3.9%; analgesic 3.9%→6.7%); the metabolic classes and the BMI gradient were unaffected (Methods, Drug classification; Results; S6 Table).

**4. Zero medications may mean no data.** *Report how many men had zero recorded medications; compare with the rest; run a sensitivity analysis excluding them; note incomplete capture.*

**Response:** Zero recorded medications occurred in 31,876 men (51.2% of the analytic cohort). Compared with medicated men they were slightly younger (72.4 vs 73.7 years) with the same BMI (23.5) and similar positivity (83.3% vs 82.8%). Facility identifiers were not available in the extract, so a facility comparison was not possible; we state this. In a sensitivity analysis restricted to users (Fig 2B), the mean number of medications was flat across the lower BMI range and rose only at high BMI, showing that the higher burden at low BMI mainly reflects a larger proportion of medicated men rather than more drugs per user. We also now state that the database captures only prescribing within participating facilities, so drugs obtained elsewhere and over-the-counter products were not observed, which may contribute to the low mean (Results; Methods; Limitations).

**5. “Polypharmacy” does not fit a mean of 1.44.** *Adopt ≥5 and ≥10 thresholds and report proportions; replace “polypharmacy” with “number of medications.”*

**Response:** We now report that 12.2% of men took five or more drugs and 2.1% took ten or more (the conventional thresholds for polypharmacy and hyperpolypharmacy). We have removed “polypharmacy” as a descriptor of the cohort's mean throughout the Abstract, Results, and Conclusion and replaced it with “number of medications.” The number of medications (count) remains the primary outcome, modeled with negative binomial regression, because it is more informative than a dichotomous threshold for a low mean count.

**6. Define the medication exposure window.** *Index date, look-back/forward interval, active vs dispensing, repeat dispensings, fixed-dose combinations, and post-biopsy prescriptions.*

**Response:** The extract contained the set of drugs recorded around the time of biopsy but did not include dispensing dates. We therefore could not define a fixed look-back or look-forward window, distinguish active from past prescriptions, or separate short-course from chronic therapy; each distinct drug was counted once per patient and fixed-dose combination products were counted as their components. We state this in the Methods (Variables) and, in the Limitations, that the counts should be read as the drugs recorded around the time of biopsy rather than a validated point-prevalence measure, and that drugs started in response to the biopsy result could not be excluded.

**7. Model BMI flexibly and report effect sizes.** *Restricted cubic splines; IRRs with CIs; absolute difference across the BMI range; bootstrap CI for the minimum; overdispersion statistic and model fit.*

**Response:** We now model BMI with a restricted cubic spline in addition to the quadratic term; the two gave a similar shape and fit (Fig 3B, revised). Overdispersion was confirmed (Poisson dispersion statistic 4.01), justifying the negative binomial model. We report incidence rate ratios with 95% CIs (BMI 0.86 [0.84–0.89]; BMI² 1.004 [1.003–1.004]), the absolute predicted counts across the range (1.50 at BMI 18, 1.47 at the minimum, 2.05 at BMI 30), and the adjusted minimum at BMI 20.5 with a bootstrap 95% CI of 19.4–21.3 (1,000 resamples). The adjusted rate was 1.40 times higher at BMI 30 than at the minimum but only 1.02 times higher at BMI 18, confirming that the increase is driven mainly by high BMI (Methods; Results; Fig 2, Fig 3).

**8. The drug-class analysis has no inferential component.** *Provide prevalence/odds ratios with CIs for each class across BMI strata, adjusted for age; state multiple-comparison handling.*

**Response:** We added age-adjusted logistic regression for each class across BMI groups (reference 20–25 kg/m²), reporting odds ratios with 95% CIs and applying a Bonferroni correction for the 20 comparisons (new S4 Table). The metabolic classes showed a clear gradient (e.g., antihypertensive/cardiovascular OR 0.79 [0.73–0.85] at BMI <20 and 1.46 [1.39–1.53] at BMI ≥25), gastrointestinal and analgesic drugs were more common at low BMI, and urological drugs changed little (not significant after correction). These results are summarized in the Results.

**9. Support the null findings with numbers.** *Odds ratios with CIs for age, BMI, and medication count; the same for the 65–75 sensitivity analysis, presented in full.*

**Response:** The multivariable logistic model is now reported in full: age OR 2.04 (95% CI 1.97–2.12), BMI 1.07 (1.03–1.11) per 5 units, and number of medications 0.87 (0.84–0.91) per 5 drugs (Fig 5D; Results; S3 Table). The 65–75-year sensitivity analysis is also reported in full (age 1.93 [1.76–2.10]; BMI 1.08 [1.03–1.13]; medications 0.83 [0.79–0.88]).

**10. PSA was collected and then not used.** *Provide the PSA-adjusted analysis in the available subgroup (as a sensitivity analysis) or a reason for omitting it; include a table comparing men with and without PSA; treat PSA absence as a first-order limitation.*

**Response:** We added the PSA-adjusted analysis as a sensitivity analysis. Among the 3,442 men (5.5% of the analytic cohort) with an available PSA, adding log-PSA left only age (OR 2.00 [1.70–2.35]) and PSA (2.12 [1.88–2.39] per log unit) associated with positivity; BMI (1.09 [0.93–1.27]) and the number of medications (0.87 [0.73–1.03]) were not. We explicitly state that, because PSA was available only in a small, selected subgroup, this does not resolve confounding by PSA. A comparison of men with and without PSA (age, BMI, medications, positivity) is provided in the new S5 Table (facility was not available). We also added, to the Limitations, that PSA—which drives both who is biopsied and who tests positive—was available in only 7.4% of the cohort and is a first-order limitation for every positivity analysis. Following Reviewer #1, we further confirmed that excluding urological drugs did not change the findings (Results).

**11. Show how the cohort was assembled.** *Total biopsies; every exclusion with counts (missing vs implausible BMI); a flow diagram; a comparison of included and excluded men.*

**Response:** We added a participant flow diagram (new S1 Fig): of 67,812 men, 5,378 with missing and 129 with implausible BMI were excluded, leaving 62,305 for the BMI-related analyses (51,754 positive, 10,551 negative). Included and excluded men were similar in age (73.1 vs 72.6 years) and positivity (83.1% vs 85.2%); the excluded men had fewer recorded medications (1.62 vs 0.87), consistent with less complete capture. These counts and the comparison are given in the Results and S1 Fig.

**12. Do not describe low BMI as frailty.** *Soften the language; state that occult illness or advanced disease could equally explain both the low BMI and the higher medication count; a cross-sectional design cannot distinguish these.*

**Response:** We removed the frailty interpretation from the Discussion and Conclusion and now describe the drug classes by name only. We added to the Limitations that we did not measure frailty, nutritional, or functional status, that occult illness or advanced disease could equally explain both a lower BMI and a higher medication count, and that our cross-sectional design cannot distinguish these possibilities.

**13. Narrow the Discussion and Conclusions.** *Shorten the material on ADT, cardiovascular risk, and fractures or present it as rationale for future work; remove the claim that the findings “may inform the care”; limit the conclusions to a descriptive profile of a biopsy-only cohort and state the selection bias.*

**Response:** We shortened the paragraph on androgen-deprivation therapy, cardiovascular risk, and fractures to three sentences and reframed it explicitly as a rationale for future work, stating that our cross-sectional data cannot establish such links. We removed “may inform the care of older men” from both the Abstract and the Conclusion; the conclusion now describes a descriptive profile of a biopsy-only cohort that is subject to selection bias and may inform the design of future risk-stratified or prospective studies rather than current clinical care.

**14. Reporting and data availability.** *Revise the Data Availability Statement to meet PLOS policy for third-party data (licensing body, contact, cost/restrictions, same-terms access). Deposit analytic code (requested).*

**Response:** We revised the Data Availability Statement (Methods statements and the submission form). The underlying raw claims data are third-party data owned by Medical Data Vision Co., Ltd. (MDV; Sumitomo Fudosan Kanda Building 10F, 7 Kanda-Mitoshirocho, Chiyoda-ku, Tokyo, Japan; tel. +81-80-6894-6061; https://www.mdv.co.jp/company/outline/), obtained under a data use agreement; the authors do not own these data and cannot redistribute the full raw database, but other researchers can obtain the same data by application to and purchase from MDV on the same terms, with no special access privileges. The de-identified analytic dataset and drug-to-class mapping used to generate the tables and figures are provided as Supporting Information (S1 File). We are also happy to deposit the analytic Python code with package versions and have prepared it for that purpose.

**Reviewer #1**

**(Strengths).** *Large real-world cohort; appropriate distinction of medication quantity from class; appropriate use of negative binomial regression; transparent about limited PSA. Exposure window needs definition; positivity validation and repeat-biopsy handling should be specified; adjustment for age alone is limited.*

**Response:** We thank the reviewer. The exposure window, positivity definition, and one-record-per-patient handling are now specified (Editor points 1 and 6). We acknowledge that adjustment was limited to age because PSA, hospital characteristics, socioeconomic factors, smoking, and documented comorbidities were unavailable or absent from the extract, and we state this in the Limitations.

**Weakness 1 (proxy; frailty not measured).** *Medication count is an imperfect proxy; single-class assignment may misclassify multi-indication drugs; the frailty interpretation is not supported.*

**Response:** Addressed. We quantified the multi-indication share (4.2%) and tested an alternative assignment rule (Editor point 3), and we removed the frailty interpretation and added the occult-illness/advanced-disease alternative (Editor point 12).

**Weakness 2 (additional analyses).** *Alternative polypharmacy definitions (≥5, ≥10); restricted cubic splines; effect sizes with CIs; PSA-adjusted subgroup; exclude urological medications; claims-based comorbidity indices and hospital-level variation.*

**Response:** We added the ≥5/≥10 proportions (Editor point 5), restricted cubic splines and effect sizes with CIs (points 7 and 8), the PSA-adjusted subgroup analysis, and the analysis excluding urological drugs (point 10). Claims-based comorbidity indices could not be constructed because diagnosis codes were absent from the extract, and hospital-level variation could not be assessed because facility identifiers were not available; we state both explicitly.

**Weakness 3 (Discussion).** *Separate descriptive associations from clinical implications; soften frailty; shorten the ADT material; emphasize that the null may be affected by limited PSA, absent stage/grade, and selection bias; temper the conclusion.*

**Response:** Addressed as described under Editor points 10, 12, and 13. The Discussion now separates associations from implications, the frailty language is softened, the ADT material is shortened to future-work rationale, the Limitations emphasize limited PSA, absent stage/grade, and selection bias, and the conclusion is limited to a descriptive profile that may inform future studies.

**Reviewer #2**

**Diagnosis data and medication-based classification.** *Absence of diagnosis codes limits interpretation of indications and the validity of medication-based classes (e.g., alpha1-blockers for BPH vs hypertension); age confounds both medication burden and prostate cancer incidence; unclear how findings advance understanding of prostate cancer development.*

**Response:** We agree and have addressed the diagnosis-data point directly (Editor point 2): diagnosis codes were absent from our extract, so a validated comorbidity index cannot be constructed and drug class is used only as an approximation. The specific ambiguity noted (uroselective α1-blockers vs doxazosin) is exactly the case we handled by predominant use and tested in the alternative-assignment sensitivity analysis (Editor point 3; S6 Table). Regarding age confounding, all key analyses are age-adjusted and the BMI–medication pattern persists within every age stratum. As the editor noted, PLOS ONE does not assess novelty or impact; the paper is presented as a descriptive medication profile of a biopsy cohort and does not claim to explain prostate cancer development.

We believe the manuscript is substantially strengthened by these revisions and thank the editor and reviewers again for their guidance.
