# Supplementary material for: Medication background of Japanese men undergoing prostate needle biopsy: A medication profile based on the number of medications and drug classes
Source: PLoS One. 2026 Oct 29;21(10):e0354283. doi: 10.1371/journal.pone.0354283 (PMC13718932; doi:10.1371/journal.pone.0354283)
Supplement: Supplementary file 7 [file pone.0354283.s011.docx]

**Response to Reviewers**

Manuscript PONE-D-26-33559R1

We thank the editor for the careful second review. We have made all the requested changes. In response to the concerns about the biopsy-positivity outcome (points 1 and 2), we have removed the positivity analyses entirely and refocused the paper on the medication background of men who undergo prostate biopsy; this also resolves the inconsistencies noted. Line numbers below refer to the clean revised manuscript.

**1. Correct the positivity conclusion.**

**Response:** We have removed all biopsy-positivity analyses from the manuscript (see point 2). The Abstract, Results, Fig 5, the S3 footnote, and the S5 Table that reported these associations have been deleted, so the manuscript no longer describes BMI or the number of medications as associated with, or independent of, cancer detection.

**2. Address the validity of the positivity outcome.**

**Response:** We agree that the claims-based positivity rate (83.2%) is implausibly high relative to Japanese biopsy series, and our extract does not contain diagnosis (disease) codes, so we cannot identify men with a prostate cancer code before biopsy or distinguish suspected from confirmed diagnoses; the most likely explanation is that negative or suspected cases were lost to follow-up at other facilities. Because the outcome cannot be validated, we have removed every positivity analysis rather than present them as exploratory: Fig 5, the S3 logistic regression (panel B), the S5 Table, the age- and PSA-stratified positivity results, and the positive/negative columns of Table 1 have all been deleted, and the positive/negative case definition has been removed from the Methods. The paper now describes a single biopsy cohort without a cancer-status outcome.

**3. Revise the BMI–medication framing.**

**Response:** We now use “U-shaped” consistently and state that the increase is driven mainly by high BMI, in the Abstract (lines 33–35), the Results, the Discussion (line 209), and the Fig 3 title (“The U-shaped association persists after age adjustment”).

**4. Align drug-class claims with S4 Table.**

**Response:** We removed the claim that psychotropic/sleep drugs are elevated at low BMI from the Abstract, Results, Discussion, and the Fig 4 legend. We now state that gastrointestinal drugs and antibiotics were elevated at both BMI extremes.

**5. Complete the cohort description.**

**Response:** All men were aged 60 years or older (range 60–99); no men younger than 60 were present, and this is now stated in the Methods and Results (line 143). S1 Fig now begins with the total number of biopsies before exclusions. We added that BMI was missing more often among men with a measured PSA than among those without (31.8% versus 6.2%; line 148), probably reflecting different recording of height and weight at the encounters where PSA was measured. We added that 31,876 men (51.2%) had no recorded medication and that about 16% of prescriptions were not assigned to any of the ten drug classes (line 150).

**6. Data and code.**

**Response:** We have added the analysis code (Python, with package versions) as Supporting Information (S2 File). Medical Data Vision Co., Ltd. permits sharing of the de-identified analytic dataset provided as S1 File, which has been re-uploaded in English with all potentially identifying fields removed (exact dates removed; age given as 5-year groups). The Data Availability statement in the submission form has been corrected to match the manuscript.

**7. Introduction frailty; title.**

**Response:** We removed the frailty wording from the Introduction (line 57) and, following the editor’s suggestion, changed “comorbidity profile” to “medication profile” in the title.

**8. Competing Interests field.**

**Response:** Corrected in the submission form to: “The authors have declared that no competing interests exist.”

**9. Human Participants Research Checklist (Questions 1–3).**

**Response:** We completed Question 3 (data accessed May 30, 2024; the authors had no access to information that could identify individual participants; Methods lines 87–91) and corrected the selections for Questions 1 (ethics approval “Uploaded”) and 2 (“N/A”, as no participants were prospectively recruited).

**10. Remove “most cases are likely to be localized.”**

**Response:** Deleted from the Limitations.

**Reference list.**

**Response:** Removing the treatment-sequelae paragraph in the previous round left nine references, all cited in the text; the list is complete and correct.

We believe the manuscript now meets PLOS ONE's criteria and thank the editor for the constructive guidance.
