# Supplementary material for: Medication background of Japanese men undergoing prostate needle biopsy: A medication profile based on the number of medications and drug classes
Source: PLoS One. 2026 Oct 29;21(10):e0354283. doi: 10.1371/journal.pone.0354283 (PMC13718932; doi:10.1371/journal.pone.0354283)
Supplement: Peer review history

# Peer review: Decision Letter 0

**DOI:** 10.1371/journal.pone.0354283.r001

**Authors:** Jabril Johnson

16 Sep 2026

PLOS One

Dear Dr. Kawahara,

Thank you for submitting your manuscript to PLOS One. After careful consideration, we believe it has merit but does not fully meet PLOS One’s publication criteria as they currently stand. Therefore, we invite you to submit a revised version of the manuscript that addresses the points raised during the review process.

==============================

**Editor Comments**

Both reviewers note the study’s scale and descriptive aim as strengths, and I agree that a claims-based profile of medication use in this population is worth publishing. The concerns below are substantial, and several require new analyses rather than rewording. I am requesting major revision.

Two points of guidance first. Reviewer #2 questions whether the findings advance understanding of prostate cancer development; PLOS ONE does not assess novelty or impact, so you need not respond to that point. Reviewer #2 also states that diagnosis data are unavailable in your dataset, while Reviewer #1 assumes a claims-based comorbidity index could be built. Settle this directly rather than answering only one reviewer.

**1. Biopsy positivity is never defined**

State whether men with a cancer code recorded before or at biopsy were retained. State how repeat biopsies in the same man were handled — "we used the record at the time of biopsy" does not say whether one man can contribute more than one record. Say whether this definition has ever been validated against pathology. If it has not, then the finding that positivity is unrelated to BMI and medication count cannot be separated from the case definition, and the manuscript must say so plainly.

**2. State what diagnosis data Medical Data Vision (MDV) contains.**

Your classification is drug-name-based, and you correctly note it only approximates disease prevalence. Could you clarify whether the diagnosis information was available and unused, or genuinely absent?

What we need: a plain statement of what diagnosis fields exist in your extract and their coding system. If diagnoses are available, explain why drug class was used as a comorbidity proxy instead, and address Reviewer #1’s request for claims-based comorbidity indices. If they are not available, say so explicitly, and state that a validated comorbidity index therefore cannot be constructed.

**3. Justify the drug classification and test it.**

The scheme in S1 Table appears reasonable, and the alpha1-blocker handling is sensible. But "the class that matched the predominant use in this population" is an assumption you have not tested.

What we need: map your classes to ATC — the WHO Anatomical Therapeutic Chemical system — so readers can audit the assignments against a published standard. Report how many drugs in your data had more than one plausible indication and what proportion of all prescriptions those represent. Rerun the class-composition analysis under at least one alternative assignment rule to show how sensitive the findings are.

**4. Zero medications may mean no data, as opposed to "no" drugs.**

Treating men with no recorded medication as taking zero drugs assumes complete capture. MDV captures prescribing within participating facilities; drugs from other providers and over-the-counter products are invisible. This assumption may well explain the low mean of 1.44.

What we need: report how many men had zero recorded medications. Compare them with the rest of the cohort on age, BMI, and facility. Run a sensitivity analysis excluding them. If capture is incomplete, the J-shaped curve may partly reflect who fills prescriptions inside the network rather than who takes medications.

**5. "Polypharmacy" does not fit a mean of 1.44 medications.**

What we need: adopt the ≥5 and ≥10 thresholds Reviewer #1 suggests, report the proportion of men meeting each, and rerun the main analyses with these as outcomes. Elsewhere, replace "polypharmacy" with "number of medications."

**6. Define the medication exposure window.**

"The record at the time of biopsy" is not a specification.

What we need: the index date; the look-back or look-forward interval in days; whether you counted active prescriptions or dispensing records; how repeat dispensings and fixed-dose combination products were counted; and whether short-course and chronic therapy were distinguished. If any medications were counted on or after the biopsy date, explain how you ruled out the diagnosis itself changing what was prescribed. Reviewer #1 raises this, and it remains unanswered.

**7. Model BMI flexibly and report effect sizes.**

A quadratic forces symmetry, so the reported minimum partly reflects that constraint rather than the data. Entering BMI and BMI² uncentered also creates collinearity.

What we need: restricted cubic splines, as Reviewer #1 requests. Report incidence rate ratios with confidence intervals and the absolute difference in medication count across the observed BMI range. With 67,812 men, p<0.001 conveys very little. If you retain the estimated minimum, give it a bootstrap confidence interval — a point estimate of 20.5 with no interval is not interpretable. Justify the negative binomial model with an overdispersion statistic and report model fit.

**8. The drug-class analysis has no inferential component.**

The 3×3 age-by-BMI grid reports prevalence only. Reviewer #1 asks for effect sizes with confidence intervals for the drug-class comparisons, and none exist.

What we need: prevalence ratios or odds ratios with confidence intervals for each class across BMI strata, adjusted for age. With ten classes across nine cells, also state how you handled multiple comparisons, or explain why the analysis should be read as purely descriptive — and if so, remove comparative language from the Results and Discussion.

**9. Support the null findings with numbers.**

The logistic model contains age, BMI, and medication count, but no estimates are reported.

What we need: odds ratios with confidence intervals for all three, so readers can tell whether an association was ruled out or merely undetected. Report the same for the 65–75 sensitivity analysis, which is a good inclusion and should be presented in full rather than mentioned.

**10. PSA was collected and then not used.**

You state that PSA availability and value were collected, but PSA doesn't appear in any model. This needs explaining.

What we need: either the PSA-adjusted analysis in the available subgroup that Reviewer #1 requests, clearly labeled as a sensitivity analysis, or a stated reason for omitting it. Either way, include a table comparing men with and without PSA data on age, BMI, facility type, and biopsy result. A 7.4% subgroup is itself selected, so do not present this as resolving confounding by PSA. Because PSA drives both who is biopsied and who tests positive, its absence is a first-order limitation for every positivity analysis and belongs in the Limitations section as such.

**11. Show how the cohort was assembled.**

You describe excluding implausible BMI values, but you don't mention how many men lacked BMI altogether.

What we need: the total number of men with a biopsy record in the study window; every exclusion with counts, separating missing BMI from implausible BMI; a participant flow diagram; and a comparison of included and excluded men. The J-shaped pattern holds only if the analytic sample resembles the men actually biopsied.

**12. Do not describe low BMI as frailty.**

You measured no frailty, nutritional, or functional status variable, as Reviewer #1 notes.

What we need: soften the language and state explicitly that occult illness or advanced disease could equally explain both the low BMI and the higher medication count. A cross-sectional design cannot distinguish these.

**13. Narrow the Discussion and Conclusions to what you studied.**

The material on androgen-deprivation therapy, cardiovascular risk, and fractures examines nothing in your dataset. Shorten it substantially or present it clearly as rationale for future work. With no treatment, stage, grade, or outcome data, the claim that the findings "may inform the care" of older men with prostate cancer cannot stand. Limit the conclusions to a descriptive profile of a biopsy-only cohort and state that cohort’s selection bias directly.

**14. Reporting and data availability.**

Ethics approval, the consent waiver, and the anonymization statement are satisfactory.

Required: the Data Availability Statement must be revised to meet PLOS policy for third-party data. Name the licensing body, give the contact information another researcher would need to apply for access, and state any cost or contractual restrictions. Confirm that other investigators can obtain the MDV extract on the same terms you did. If they cannot, PLOS policy requires a supporting analysis of publicly available data that validates your conclusions. A statement that the data are proprietary is not sufficient.

Requested: Given the number of analytic decisions that are currently undocumented, I would also ask you to deposit your analytic code with Python and package versions, though this is a request rather than a condition.

Please submit a point-by-point response showing where each change appears in the revised manuscript.

==============================

Please submit your revised manuscript by Nov 15 2026 11:59PM. If you will need more time than this to complete your revisions, please reply to this message or contact the journal office at plosone@plos.org. When you're ready to submit your revision, log on to https://www.editorialmanager.com/pone/ and select the 'Submissions Needing Revision' folder to locate your manuscript file.

- A letter that responds to each point raised by the academic editor and reviewer(s). You should upload this letter as a separate file labeled 'Response to Reviewers'.
- A marked-up copy of your manuscript that highlights changes made to the original version. Upload this as a separate file labeled 'Revised Manuscript with Track Changes'.
- An unmarked version of your revised paper without tracked changes. Upload this as a separate file labeled 'Manuscript'.

If you would like to make changes to your financial disclosure, please include your updated statement in your cover letter. Guidelines for resubmitting your figure files are available below the reviewer comments at the end of this letter.

If applicable, we recommend that you deposit your laboratory protocols in protocols.io to enhance the reproducibility of your results. Protocols.io assigns your protocol its own identifier (DOI) so that it can be cited independently in the future. For instructions, see: https://journals.plos.org/plosone/s/submission-guidelines#loc-laboratory-protocols. Additionally, PLOS One offers an option for publishing peer-reviewed Lab Protocol articles, which describe protocols hosted on protocols.io. Read more information on sharing protocols at https://plos.org/protocols?utm_medium=editorial-email&utm_source=authorletters&utm_campaign=protocols.

As the corresponding author, your ORCID iD is verified in the submission system and will appear in the published article. PLOS supports ORCID, and we encourage all coauthors to register for an ORCID iD and use it. Please encourage your coauthors to verify their ORCID iD within the submission system before final acceptance, as unverified ORCID iDs will not appear in the published article. *Only*  the individual author can complete the verification step; PLOS staff *cannot*  verify ORCID iDs on behalf of authors.

We look forward to receiving your revised manuscript.

Kind regards,

Jabril Johnson, Ph.D.

Guest Editor

PLOS One

Journal Requirements:

When submitting your revision, we need you to address these additional requirements.

1. Please ensure that your manuscript meets PLOS ONE’s style requirements, including those for file naming. The PLOS ONE style templates can be found at https://journals.plos.org/plosone/s/file?id=wjVg/PLOSOne_formatting_sample_main_body.pdf and https://journals.plos.org/plosone/s/file?id=ba62/PLOSOne_formatting_sample_title_authors_affiliations.pdf

If the reviewer comments include a recommendation to cite specific previously published works, please review and evaluate these publications to determine whether they are relevant and should be cited. There is no requirement to cite these works unless the editor has indicated otherwise.

[Note: HTML markup is below. Please do not edit.]

Reviewers' comments:

Reviewer’s Responses to Questions

**Comments to the Author**

1. Is the manuscript technically sound, and do the data support the conclusions?

Reviewer #1: Yes

Reviewer #2: No

**********

2. Has the statistical analysis been performed appropriately and rigorously?

Reviewer #1: Yes

Reviewer #2: I Don't Know

**********

3. Have the authors made all data underlying the findings in their manuscript fully available?

The PLOS Data policy

Reviewer #1: Yes

Reviewer #2: Yes

**********

4. Is the manuscript presented in an intelligible fashion and written in standard English?

Reviewer #1: Yes

Reviewer #2: Yes

**********

Reviewer #1: Summary:

This large real-world cohort of 67,812 Japanese men undergoing prostate biopsy is a major strength. The study addresses an understudied but clinically relevant issue: medication burden and medication-class patterns at the time of prostate cancer evaluation. The analysis appropriately distinguishes medication quantity from medication class, demonstrates a consistent age-adjusted J-shaped association between BMI and medication count, and transparently acknowledges the limited PSA availability. The retrospective claims-based design is suitable for descriptive epidemiology, and the use of negative binomial regression for overdispersed medication counts is appropriate. However, the medication-exposure window at the time of biopsy needs explicit definition (e.g., active prescription, dispensing period, or look-back interval). The manuscript should also specify how biopsy positivity was validated from claims data and whether repeated biopsies for the same individual were excluded. Adjustment for age alone is limited; important confounders, including PSA, hospital characteristics, socioeconomic factors, smoking, and documented comorbidities, were unavailable or not included.

Weaknesses:

There are a couple of weaknesses:

1- Medication count is an imperfect proxy for comorbidity because over-the-counter medications, adherence, dose, treatment duration, and medication indications were unavailable. Assigning each drug to one “predominant” class may also misclassify drugs with multiple indications. The suggestion that low-BMI medication patterns indicate frailty is plausible but not supported by direct frailty, nutritional, functional-status, or diagnostic-comorbidity measures.

2-Experiments: Additional analyses are needed rather than new experiments. The authors should perform sensitivity analyses using alternative definitions of polypharmacy (e.g., ≥5 and ≥10 concurrent medications), model BMI flexibly with restricted cubic splines rather than only a quadratic term, and report effect sizes with confidence intervals for all key drug-class comparisons. Analyses should also adjust for PSA levels in the available subgroup and evaluate whether findings persist after excluding urological medications, which may be directly related to biopsy referral. If feasible, incorporating claims-based comorbidity indices and evaluating hospital-level variation would strengthen interpretation. New prospective studies with validated medication exposure, direct comorbidity and frailty measures, PSA, tumor stage/grade, and treatment outcomes are needed to confirm the clinical relevance of these findings.

3-As to discussion, after thorough reading, the PIs separate descriptive associations from clinical implications. The findings support that medication burden and drug-class profiles vary by BMI and age among men undergoing biopsy; they do not establish that these patterns influence treatment selection, treatment tolerability, prognosis, or cancer detection. Statements suggesting that low BMI reflects frailty should be softened because frailty was not measured. Similarly, the extended discussion of androgen-deprivation therapy, cardiovascular risk, and fractures is clinically relevant background. It isn't directly evaluated in this dataset; please shorten it or present it as a rationale for future research. In addition, they should emphasize that the null association between BMI and medication count and biopsy positivity may be affected by limited PSA data (available in only 7.4%), the absence of tumor stage/grade, and selection bias inherent to a biopsy-only cohort. The conclusion should avoid stating that the results “may help in the care” of older men with prostate cancer unless the authors specify that the study provides descriptive information for future risk-stratified or prospective studies.

Reviewer #2: While the study highlights the high prevalence of medication use and polypharmacy among men undergoing prostate biopsy, the absence of diagnosis data (e.g., akin to ICD-10 codes used in the United States of America) limits the interpretation of medication indications and the validity of medication-based classifications. For example, alpha1-blockers may be prescribed for benign prostatic hyperplasia or hypertension, making it inappropriate to infer underlying clinical conditions without access to diagnostic information from the medical record. Furthermore, because both medication burden and prostate cancer incidence increase with age, the observed associations are not unexpected, and it remains unclear how these findings advance our understanding of environmental or medication-related influences on prostate cancer development.

**********

what does this mean?). If published, this will include your full peer review and any attached files.

If you choose “no”, your identity will remain anonymous, but your review may still be made public.

**Do you want your identity to be public for this peer review?**  For information about this choice, including consent withdrawal, please see our Privacy Policy

Reviewer #1: **Yes:**  Firas Kobeissy

Reviewer #2: No

**********

[NOTE: If reviewer comments were submitted as an attachment file, they will be attached to this email and accessible via the submission site. Please log into your account, locate the manuscript record, and check for the action link "View Attachments". If this link does not appear, there are no attachment files.]

To ensure your figures meet our technical requirements, please review our figure guidelines: https://journals.plos.org/plosone/s/figures

You may also use PLOS’s free figure tool, NAAS, to help you prepare p figures: https://journals.plos.org/plosone/s/figures#loc-tools-for-figure-preparation.

NAAS will assess whether your figures meet our technical requirements by comparing each figure against our figure specifications.

# Author comment: Author response to Decision Letter 1

**DOI:** 10.1371/journal.pone.0354283.r002

22 Sep 2026

Response to Reviewers

Manuscript PONE-D-26-33559

Medication background of Japanese men undergoing prostate needle biopsy: a comorbidity profile based on the number of medications and drug classes

We thank the editor and both reviewers for their careful and constructive assessment. We have revised the manuscript substantially, including several new analyses. Below we respond to each point in turn and indicate where the corresponding change appears in the revised manuscript. Revisions are shown in the marked-up manuscript; line numbers refer to the clean revised version. New Supporting Information items (S1 Fig; S4, S5, and S6 Tables) have been added.

Editor — general guidance

Novelty/impact (raised by Reviewer #2). Whether the findings advance understanding of prostate cancer development.

Response: We note the editor’s guidance that PLOS ONE does not assess novelty or impact, and we have not framed the paper around advancing the understanding of prostate cancer development. The study is presented as a descriptive profile of the medication background of men undergoing prostate biopsy.

Diagnosis data — settle directly. Reviewer #2 states diagnosis data are unavailable; Reviewer #1 assumes a claims-based comorbidity index could be built.

Response: We have settled this directly. Our extract from the Medical Data Vision (MDV) database did not include diagnosis (disease) codes; it contained the prescribed drug names, demographics, BMI, and PSA availability/value only. We therefore could not identify comorbidities directly or construct a validated claims-based comorbidity index (e.g., a Charlson or Elixhauser index), and we used drug classes as an approximate marker of the underlying conditions. This is now stated explicitly in the Methods (Drug classification) and in the Limitations.

Editor — specific points

1. Biopsy positivity definition. State whether men with a cancer code before/at biopsy were retained, how repeat biopsies were handled, and whether the definition was validated against pathology.

Response: Positivity was defined from the claims-based prostate cancer classification recorded in the database and was not validated against pathology reports, which were not available. Men with a prostate cancer code recorded before biopsy were not separately excluded. The analytic dataset contains one record per patient (each identifier appears once; no patient appears in both groups), so repeat biopsies do not contribute more than one observation. We now state this in the Methods (Study design and data source) and add, in the Limitations, that because the case definition is unvalidated, the finding that positivity is unrelated to BMI and to the number of medications cannot be fully separated from that definition.

2. What diagnosis data MDV contains. State whether diagnosis information was available and unused, or genuinely absent.

Response: As above, diagnosis codes were genuinely absent from our extract. We state that they were not included, that a validated comorbidity index therefore cannot be constructed, and that drug class was used as an approximation (Methods, Drug classification; Limitations).

3. Justify and test the drug classification (ATC; multi-indication drugs; alternative rule). Map classes to ATC; report how many drugs had more than one plausible indication; rerun under an alternative assignment rule.

Response: We mapped each therapeutic class to the corresponding ATC groups (new S6 Table) so the assignments can be audited against a published standard. Drugs with more than one plausible indication accounted for only 4.2% of all prescriptions. In a sensitivity analysis using an alternative rule (low-dose aspirin reassigned to analgesic, doxazosin to urological), the prevalence of every class was essentially unchanged except for the two deliberately reassigned classes (antithrombotic 6.0%→3.9%; analgesic 3.9%→6.7%); the metabolic classes and the BMI gradient were unaffected (Methods, Drug classification; Results; S6 Table).

4. Zero medications may mean no data. Report how many men had zero recorded medications; compare with the rest; run a sensitivity analysis excluding them; note incomplete capture.

Response: Zero recorded medications occurred in 31,876 men (51.2% of the analytic cohort). Compared with medicated men they were slightly younger (72.4 vs 73.7 years) with the same BMI (23.5) and similar positivity (83.3% vs 82.8%). Facility identifiers were not available in the extract, so a facility comparison was not possible; we state this. In a sensitivity analysis restricted to users (Fig 2B), the mean number of medications was flat across the lower BMI range and rose only at high BMI, showing that the higher burden at low BMI mainly reflects a larger proportion of medicated men rather than more drugs per user. We also now state that the database captures only prescribing within participating facilities, so drugs obtained elsewhere and over-the-counter products were not observed, which may contribute to the low mean (Results; Methods; Limitations).

5. “Polypharmacy” does not fit a mean of 1.44. Adopt ≥5 and ≥10 thresholds and report proportions; replace “polypharmacy” with “number of medications.”

Response: We now report that 12.2% of men took five or more drugs and 2.1% took ten or more (the conventional thresholds for polypharmacy and hyperpolypharmacy). We have removed “polypharmacy” as a descriptor of the cohort’s mean throughout the Abstract, Results, and Conclusion and replaced it with “number of medications.” The number of medications (count) remains the primary outcome, modeled with negative binomial regression, because it is more informative than a dichotomous threshold for a low mean count.

6. Define the medication exposure window. Index date, look-back/forward interval, active vs dispensing, repeat dispensings, fixed-dose combinations, and post-biopsy prescriptions.

Response: The extract contained the set of drugs recorded around the time of biopsy but did not include dispensing dates. We therefore could not define a fixed look-back or look-forward window, distinguish active from past prescriptions, or separate short-course from chronic therapy; each distinct drug was counted once per patient and fixed-dose combination products were counted as their components. We state this in the Methods (Variables) and, in the Limitations, that the counts should be read as the drugs recorded around the time of biopsy rather than a validated point-prevalence measure, and that drugs started in response to the biopsy result could not be excluded.

7. Model BMI flexibly and report effect sizes. Restricted cubic splines; IRRs with CIs; absolute difference across the BMI range; bootstrap CI for the minimum; overdispersion statistic and model fit.

Response: We now model BMI with a restricted cubic spline in addition to the quadratic term; the two gave a similar shape and fit (Fig 3B, revised). Overdispersion was confirmed (Poisson dispersion statistic 4.01), justifying the negative binomial model. We report incidence rate ratios with 95% CIs (BMI 0.86 [0.84–0.89]; BMI² 1.004 [1.003–1.004]), the absolute predicted counts across the range (1.50 at BMI 18, 1.47 at the minimum, 2.05 at BMI 30), and the adjusted minimum at BMI 20.5 with a bootstrap 95% CI of 19.4–21.3 (1,000 resamples). The adjusted rate was 1.40 times higher at BMI 30 than at the minimum but only 1.02 times higher at BMI 18, confirming that the increase is driven mainly by high BMI (Methods; Results; Fig 2, Fig 3).

8. The drug-class analysis has no inferential component. Provide prevalence/odds ratios with CIs for each class across BMI strata, adjusted for age; state multiple-comparison handling.

Response: We added age-adjusted logistic regression for each class across BMI groups (reference 20–25 kg/m²), reporting odds ratios with 95% CIs and applying a Bonferroni correction for the 20 comparisons (new S4 Table). The metabolic classes showed a clear gradient (e.g., antihypertensive/cardiovascular OR 0.79 [0.73–0.85] at BMI <20 and 1.46 [1.39–1.53] at BMI ≥25), gastrointestinal and analgesic drugs were more common at low BMI, and urological drugs changed little (not significant after correction). These results are summarized in the Results.

9. Support the null findings with numbers. Odds ratios with CIs for age, BMI, and medication count; the same for the 65–75 sensitivity analysis, presented in full.

Response: The multivariable logistic model is now reported in full: age OR 2.04 (95% CI 1.97–2.12), BMI 1.07 (1.03–1.11) per 5 units, and number of medications 0.87 (0.84–0.91) per 5 drugs (Fig 5D; Results; S3 Table). The 65–75-year sensitivity analysis is also reported in full (age 1.93 [1.76–2.10]; BMI 1.08 [1.03–1.13]; medications 0.83 [0.79–0.88]).

10. PSA was collected and then not used. Provide the PSA-adjusted analysis in the available subgroup (as a sensitivity analysis) or a reason for omitting it; include a table comparing men with and without PSA; treat PSA absence as a first-order limitation.

Response: We added the PSA-adjusted analysis as a sensitivity analysis. Among the 3,442 men (5.5% of the analytic cohort) with an available PSA, adding log-PSA left only age (OR 2.00 [1.70–2.35]) and PSA (2.12 [1.88–2.39] per log unit) associated with positivity; BMI (1.09 [0.93–1.27]) and the number of medications (0.87 [0.73–1.03]) were not. We explicitly state that, because PSA was available only in a small, selected subgroup, this does not resolve confounding by PSA. A comparison of men with and without PSA (age, BMI, medications, positivity) is provided in the new S5 Table (facility was not available). We also added, to the Limitations, that PSA—which drives both who is biopsied and who tests positive—was available in only 7.4% of the cohort and is a first-order limitation for every positivity analysis. Following Reviewer #1, we further confirmed that excluding urological drugs did not change the findings (Results).

11. Show how the cohort was assembled. Total biopsies; every exclusion with counts (missing vs implausible BMI); a flow diagram; a comparison of included and excluded men.

Response: We added a participant flow diagram (new S1 Fig): of 67,812 men, 5,378 with missing and 129 with implausible BMI were excluded, leaving 62,305 for the BMI-related analyses (51,754 positive, 10,551 negative). Included and excluded men were similar in age (73.1 vs 72.6 years) and positivity (83.1% vs 85.2%); the excluded men had fewer recorded medications (1.62 vs 0.87), consistent with less complete capture. These counts and the comparison are given in the Results and S1 Fig.

12. Do not describe low BMI as frailty. Soften the language; state that occult illness or advanced disease could equally explain both the low BMI and the higher medication count; a cross-sectional design cannot distinguish these.

Response: We removed the frailty interpretation from the Discussion and Conclusion and now describe the drug classes by name only. We added to the Limitations that we did not measure frailty, nutritional, or functional status, that occult illness or advanced disease could equally explain both a lower BMI and a higher medication count, and that our cross-sectional design cannot distinguish these possibilities.

13. Narrow the Discussion and Conclusions. Shorten the material on ADT, cardiovascular risk, and fractures or present it as rationale for future work; remove the claim that the findings “may inform the care”; limit the conclusions to a descriptive profile of a biopsy-only cohort and state the selection bias.

Response: We shortened the paragraph on androgen-deprivation therapy, cardiovascular risk, and fractures to three sentences and reframed it explicitly as a rationale for future work, stating that our cross-sectional data cannot establish such links. We removed “may inform the care of older men” from both the Abstract and the Conclusion; the conclusion now describes a descriptive profile of a biopsy-only cohort that is subject to selection bias and may inform the design of future risk-stratified or prospective studies rather than current clinical care.

14. Reporting and data availability. Revise the Data Availability Statement to meet PLOS policy for third-party data (licensing body, contact, cost/restrictions, same-terms access). Deposit analytic code (requested).

Response: We revised the Data Availability Statement (Methods statements and the submission form). The underlying raw claims data are third-party data owned by Medical Data Vision Co., Ltd. (MDV; Sumitomo Fudosan Kanda Building 10F, 7 Kanda-Mitoshirocho, Chiyoda-ku, Tokyo, Japan; tel. +81-80-6894-6061; https://www.mdv.co.jp/company/outline/), obtained under a data use agreement; the authors do not own these data and cannot redistribute the full raw database, but other researchers can obtain the same data by application to and purchase from MDV on the same terms, with no special access privileges. The de-identified analytic dataset and drug-to-class mapping used to generate the tables and figures are provided as Supporting Information (S1 File). We are also happy to deposit the analytic Python code with package versions and have prepared it for that purpose.

Reviewer #1

(Strengths). Large real-world cohort; appropriate distinction of medication quantity from class; appropriate use of negative binomial regression; transparent about limited PSA. Exposure window needs definition; positivity validation and repeat-biopsy handling should be specified; adjustment for age alone is limited.

Response: We thank the reviewer. The exposure window, positivity definition, and one-record-per-patient handling are now specified (Editor points 1 and 6). We acknowledge that adjustment was limited to age because PSA, hospital characteristics, socioeconomic factors, smoking, and documented comorbidities were unavailable or absent from the extract, and we state this in the Limitations.

Weakness 1 (proxy; frailty not measured). Medication count is an imperfect proxy; single-class assignment may misclassify multi-indication drugs; the frailty interpretation is not supported.

Response: Addressed. We quantified the multi-indication share (4.2%) and tested an alternative assignment rule (Editor point 3), and we removed the frailty interpretation and added the occult-illness/advanced-disease alternative (Editor point 12).

Weakness 2 (additional analyses). Alternative polypharmacy definitions (≥5, ≥10); restricted cubic splines; effect sizes with CIs; PSA-adjusted subgroup; exclude urological medications; claims-based comorbidity indices and hospital-level variation.

Response: We added the ≥5/≥10 proportions (Editor point 5), restricted cubic splines and effect sizes with CIs (points 7 and 8), the PSA-adjusted subgroup analysis, and the analysis excluding urological drugs (point 10). Claims-based comorbidity indices could not be constructed because diagnosis codes were absent from the extract, and hospital-level variation could not be assessed because facility identifiers were not available; we state both explicitly.

Weakness 3 (Discussion). Separate descriptive associations from clinical implications; soften frailty; shorten the ADT material; emphasize that the null may be affected by limited PSA, absent stage/grade, and selection bias; temper the conclusion.

Response: Addressed as described under Editor points 10, 12, and 13. The Discussion now separates associations from implications, the frailty language is softened, the ADT material is shortened to future-work rationale, the Limitations emphasize limited PSA, absent stage/grade, and selection bias, and the conclusion is limited to a descriptive profile that may inform future studies.

Reviewer #2

Diagnosis data and medication-based classification. Absence of diagnosis codes limits interpretation of indications and the validity of medication-based classes (e.g., alpha1-blockers for BPH vs hypertension); age confounds both medication burden and prostate cancer incidence; unclear how findings advance understanding of prostate cancer development.

Response: We agree and have addressed the diagnosis-data point directly (Editor point 2): diagnosis codes were absent from our extract, so a validated comorbidity index cannot be constructed and drug class is used only as an approximation. The specific ambiguity noted (uroselective α1-blockers vs doxazosin) is exactly the case we handled by predominant use and tested in the alternative-assignment sensitivity analysis (Editor point 3; S6 Table). Regarding age confounding, all key analyses are age-adjusted and the BMI–medication pattern persists within every age stratum. As the editor noted, PLOS ONE does not assess novelty or impact; the paper is presented as a descriptive medication profile of a biopsy cohort and does not claim to explain prostate cancer development.

We believe the manuscript is substantially strengthened by these revisions and thank the editor and reviewers again for their guidance.

Attachment Submitted filename: Response_to_Reviewers.docx

# Editor report: Decision Letter 1

**DOI:** 10.1371/journal.pone.0354283.r003

**Authors:** Jabril Johnson

2 Oct 2026

Medication background of Japanese men undergoing prostate needle biopsy: a comorbidity profile based on the number of medications and drug classes

PLOS One

Dear Dr. Kawahara,

Thank you for submitting your manuscript to PLOS One. After careful consideration, we feel that it has merit but does not fully meet PLOS One’s publication criteria as it currently stands. Therefore, we invite you to submit a revised version of the manuscript that addresses the points raised during the review process.

==============================

1. **Correct the positivity conclusion.**  Your own results show statistically significant associations with positivity for BMI (OR 1.07 per 5 units, 95% CI 1.03–1.11) and for the number of medications (OR 0.87 per 5 drugs, 95% CI 0.84–0.91; S3 Table). The same holds in the 65–75-year subgroup and after excluding urological drugs. Please revise the Abstract (lines 40–45), the Results (lines 219–222, 225–228, and 231–232), Fig 5, and the S3 footnote, and report the CIs in the text. If you consider these effects clinically unimportant, say so explicitly rather than describing them as absent.
2. **Address the validity of the positivity outcome.**  The positivity rates (83.2% overall; 59.1% at PSA <4 ng/mL) are roughly twice the approximately 40% detection rate reported in Japanese biopsy series and guidelines. Please explain how cancer status was derived from the claims data, including whether suspected-diagnosis codes, or codes recorded before or after the biopsy, were counted as positive. If men with a prostate cancer code before biopsy can be identified, please repeat the analyses excluding them. If not, please present the positivity analyses as exploratory and remove them from the Abstract’s conclusions.
3. **Revise the BMI–medication framing.**  The adjusted rate at BMI 18 is only 1.02 times the minimum, and the count among users is flat across the lower BMI range. The increase is therefore driven by high BMI. Please revise the Abstract (lines 33–35), the Discussion (lines 236–237), and the Fig 3 title accordingly, and use one term (U- or J-shaped) consistently.
4. **Align drug-class claims with S4 Table.**  Psychotropic/sleep drugs at BMI <20 do not survive your Bonferroni correction. Please remove this claim from lines 39, 198, 203, 241, and 290. Please also note that gastrointestinal drugs and antibiotics are elevated at both BMI extremes.
5. **Complete the cohort description.**  State whether men under 60 were excluded (none appear in S1 File), and add total biopsies before exclusions to S1 Fig. Explain why BMI is missing for 31.8% of PSA-tested men, compared with 6.2% of men without PSA. Add the zero-medication data (n = 31,876) to the Results; the response letter says this was done, but it is not in the text. State that about 16% of prescriptions were not assigned to a drug class.
6. **Data and code.**  Deposit the analysis code in a public repository with a persistent identifier. Confirm that MDV permits public sharing of the S1 File dataset. Correct the Data Availability field in the submission form, which contradicts the manuscript.
7. Remove the frailty framing in the Introduction (lines 60–61), and consider replacing "comorbidity profile" in the title with "medication profile."
8. Correct the Competing Interests field in the submission form, which contains the ethics statement.
9. In the Human Participants Research Checklist, complete Question 3 (lines 89–96) and correct the selections for Questions 1 and 2.
10. Please also remove lines 263–264 ("most cases are likely to be localized").
11. Provide line numbers for every change in the response letter

==============================

Please submit your revised manuscript by Nov 16 2026 11:59PM. If you will need more time than this to complete your revisions, please reply to this message or contact the journal office at plosone@plos.org. When you're ready to submit your revision, log on to https://www.editorialmanager.com/pone/ and select the 'Submissions Needing Revision' folder to locate your manuscript file.

Please include the following items when submitting your revised manuscript:

- A letter that responds to each point raised by the academic editor and reviewer(s). You should upload this letter as a separate file labeled 'Response to Reviewers'.
- A marked-up copy of your manuscript that highlights changes made to the original version. You should upload this as a separate file labeled 'Revised Manuscript with Track Changes'.
- An unmarked version of your revised paper without tracked changes. You should upload this as a separate file labeled 'Manuscript'.

If you would like to make changes to your financial disclosure, please include your updated statement in your cover letter. Guidelines for resubmitting your figure files are available below the reviewer comments at the end of this letter.

If applicable, we recommend that you deposit your laboratory protocols in protocols.io to enhance the reproducibility of your results. Protocols.io assigns your protocol its own identifier (DOI) so that it can be cited independently in the future. For instructions see: https://journals.plos.org/plosone/s/submission-guidelines#loc-laboratory-protocols. Additionally, PLOS One offers an option for publishing peer-reviewed Lab Protocol articles, which describe protocols hosted on protocols.io. Read more information on sharing protocols at https://plos.org/protocols?utm_medium=editorial-email&utm_source=authorletters&utm_campaign=protocols.

As the corresponding author, your ORCID iD is verified in the submission system and will appear in the published article. PLOS supports the use of ORCID, and we encourage all coauthors to register for an ORCID iD and use it as well. Please encourage your coauthors to verify their ORCID iD within the submission system before final acceptance, as unverified ORCID iDs will not appear in the published article. *Only*  the individual author can complete the verification step; PLOS staff *cannot*  verify ORCID iDs on behalf of authors.

We look forward to receiving your revised manuscript.

Kind regards,

Jabril Johnson, Ph.D.

Guest Editor

PLOS One

Journal Requirements:

If the reviewer comments include a recommendation to cite specific previously published works, please review and evaluate these publications to determine whether they are relevant and should be cited. There is no requirement to cite these works unless the editor has indicated otherwise.

Please review your reference list to ensure that it is complete and correct. If you have cited papers that have been retracted, please include the rationale for doing so in the manuscript text, or remove these references and replace them with relevant current references. Any changes to the reference list should be mentioned in the rebuttal letter that accompanies your revised manuscript. If you need to cite a retracted article, indicate the article’s retracted status in the References list and also include a citation and full reference for the retraction notice.

[Note: HTML markup is below. Please do not edit.]

To ensure your figures meet our technical requirements, please review our figure guidelines: https://journals.plos.org/plosone/s/figures

You may also use PLOS’s free figure tool, NAAS, to help you prepare publication quality figures: https://journals.plos.org/plosone/s/figures#loc-tools-for-figure-preparation.

NAAS will assess whether your figures meet our technical requirements by comparing each figure against our figure specifications.

# Author comment: Author response to Decision Letter 2

**DOI:** 10.1371/journal.pone.0354283.r004

5 Oct 2026

Response to Reviewers

Manuscript PONE-D-26-33559R1

We thank the editor for the careful second review. We have made all the requested changes. In response to the concerns about the biopsy-positivity outcome (points 1 and 2), we have removed the positivity analyses entirely and refocused the paper on the medication background of men who undergo prostate biopsy; this also resolves the inconsistencies noted. Line numbers below refer to the clean revised manuscript.

1. Correct the positivity conclusion.

Response: We have removed all biopsy-positivity analyses from the manuscript (see point 2). The Abstract, Results, Fig 5, the S3 footnote, and the S5 Table that reported these associations have been deleted, so the manuscript no longer describes BMI or the number of medications as associated with, or independent of, cancer detection.

2. Address the validity of the positivity outcome.

Response: We agree that the claims-based positivity rate (83.2%) is implausibly high relative to Japanese biopsy series, and our extract does not contain diagnosis (disease) codes, so we cannot identify men with a prostate cancer code before biopsy or distinguish suspected from confirmed diagnoses; the most likely explanation is that negative or suspected cases were lost to follow-up at other facilities. Because the outcome cannot be validated, we have removed every positivity analysis rather than present them as exploratory: Fig 5, the S3 logistic regression (panel B), the S5 Table, the age- and PSA-stratified positivity results, and the positive/negative columns of Table 1 have all been deleted, and the positive/negative case definition has been removed from the Methods. The paper now describes a single biopsy cohort without a cancer-status outcome.

3. Revise the BMI–medication framing.

Response: We now use “U-shaped” consistently and state that the increase is driven mainly by high BMI, in the Abstract (lines 33–35), the Results, the Discussion (line 209), and the Fig 3 title (“The U-shaped association persists after age adjustment”).

4. Align drug-class claims with S4 Table.

Response: We removed the claim that psychotropic/sleep drugs are elevated at low BMI from the Abstract, Results, Discussion, and the Fig 4 legend. We now state that gastrointestinal drugs and antibiotics were elevated at both BMI extremes.

5. Complete the cohort description.

Response: All men were aged 60 years or older (range 60–99); no men younger than 60 were present, and this is now stated in the Methods and Results (line 143). S1 Fig now begins with the total number of biopsies before exclusions. We added that BMI was missing more often among men with a measured PSA than among those without (31.8% versus 6.2%; line 148), probably reflecting different recording of height and weight at the encounters where PSA was measured. We added that 31,876 men (51.2%) had no recorded medication and that about 16% of prescriptions were not assigned to any of the ten drug classes (line 150).

6. Data and code.

Response: We have added the analysis code (Python, with package versions) as Supporting Information (S2 File). Medical Data Vision Co., Ltd. permits sharing of the de-identified analytic dataset provided as S1 File, which has been re-uploaded in English with all potentially identifying fields removed (exact dates removed; age given as 5-year groups). The Data Availability statement in the submission form has been corrected to match the manuscript.

7. Introduction frailty; title.

Response: We removed the frailty wording from the Introduction (line 57) and, following the editor’s suggestion, changed “comorbidity profile” to “medication profile” in the title.

8. Competing Interests field.

Response: Corrected in the submission form to: “The authors have declared that no competing interests exist.”

9. Human Participants Research Checklist (Questions 1–3).

Response: We completed Question 3 (data accessed May 30, 2024; the authors had no access to information that could identify individual participants; Methods lines 87–91) and corrected the selections for Questions 1 (ethics approval “Uploaded”) and 2 (“N/A”, as no participants were prospectively recruited).

10. Remove “most cases are likely to be localized.”

Response: Deleted from the Limitations.

Reference list.

Response: Removing the treatment-sequelae paragraph in the previous round left nine references, all cited in the text; the list is complete and correct.

We believe the manuscript now meets PLOS ONE’s criteria and thank the editor for the constructive guidance.

Attachment Submitted filename: Response_to_Reviewers_R2.docx

# Editor report: Decision Letter 2

**DOI:** 10.1371/journal.pone.0354283.r005

**Authors:** Jabril Johnson

12 Oct 2026

Medication background of Japanese men undergoing prostate needle biopsy: a medication profile based on the number of medications and drug classes

PONE-D-26-33559R2

Dear Dr. Kawahara,

We’re pleased to inform you that your manuscript has been judged scientifically suitable for publication and will be formally accepted for publication once it meets all outstanding technical requirements.

Within one week, you’ll receive an e-mail detailing the required amendments. When these have been addressed, you’ll receive a formal acceptance letter and your manuscript will be scheduled for publication.

An invoice will be generated when your article is formally accepted. Please note, if your institution has a publishing partnership with PLOS and your article meets the relevant criteria, all or part of your publication costs will be covered. Please make sure your user information is up-to-date by logging into Editorial Manager at Editorial Manager® and clicking the ‘Update My Information' link at the top of the page. For questions related to billing, please contact billing support.

If your institution or institutions have a press office, please notify them about your upcoming paper to help maximize its impact. If they’ll be preparing press materials, please inform our press team as soon as possible -- no later than 48 hours after receiving the formal acceptance. Your manuscript will remain under strict press embargo until 2 pm Eastern Time on the date of publication. For more information, please contact onepress@plos.org.

Kind regards,

Jabril Johnson, Ph.D.

Guest Editor

PLOS One

Additional Editor Comments (optional):

Reviewers' comments:

# Editor report: Acceptance letter

**DOI:** 10.1371/journal.pone.0354283.r006

**Authors:** Jabril Johnson

PONE-D-26-33559R2

PLOS One

Dear Dr. Kawahara,

I'm pleased to inform you that your manuscript has been deemed suitable for publication in PLOS One. Congratulations! Your manuscript is now being handed over to our production team.

At this stage, our production department will prepare your paper for publication. This includes ensuring the following:

* All references, tables, and figures are properly cited

* All relevant supporting information is included in the manuscript submission,

* There are no issues that prevent the paper from being properly typeset

You will receive further instructions from the production team, including instructions on how to review your proof when it is ready. Please keep in mind that we are working through a large volume of accepted articles, so please give us a few days to review your paper and let you know the next and final steps.

Lastly, if your institution or institutions have a press office, please let them know about your upcoming paper now to help maximize its impact. If they'll be preparing press materials, please inform our press team within the next 48 hours. Your manuscript will remain under strict press embargo until 2 pm Eastern Time on the date of publication. For more information, please contact onepress@plos.org.

You will receive an invoice from PLOS for your publication fee after your manuscript has reached the completed accept phase. If you receive an email requesting payment before acceptance or for any other service, this may be a phishing scheme. Learn how to identify phishing emails and protect your accounts at https://explore.plos.org/phishing.

If we can help with anything else, please email us at customercare@plos.org.

Thank you for submitting your work to PLOS One and supporting open access.

Kind regards,

PLOS One Editorial Office Staff

on behalf of

Dr. Jabril Johnson

Guest Editor

PLOS One
